# Status of insecticide resistance in *Anopheles gambiae* (*s.l*.) of The Gambia

**DOI:** 10.1186/s13071-019-3538-0

**Published:** 2019-06-04

**Authors:** Kevin Ochieng’ Opondo, Musa Jawara, Saihou Cham, Ebrima Jatta, Lamin Jarju, Muhammed Camara, Fatou Sanneh, Pa Modou Gaye, Lamin Jadama, Sainey Ceesay, Ebrima Njie, Benoit Sessinou Assogba, Balla Kandeh, Umberto D’Alessandro

**Affiliations:** 1 Medical Research Council Unit The Gambia at the London School of Hygiene and Tropical Medicine, Banjul, The Gambia; 2 School of Arts and Sciences, University of Gambia, Banjul, The Gambia; 3 Ministry of Health, The Gambia National Malaria Control Programme, Banjul, The Gambia

**Keywords:** *Anopheles gambiae*, Insecticide resistance, Insecticide resistance management, *kdr*, *Ace-1*, Malaria

## Abstract

**Background:**

Vector control activities, namely long-lasting insecticidal nets (LLIN) and indoor residual spraying (IRS), have contributed significantly to the decreasing malaria burden observed in The Gambia since 2008. Nevertheless, insecticide resistance may threaten such success; it is important to regularly assess the susceptibility of local malaria vectors to available insecticides.

**Methods:**

In the transmission seasons of 2016 and 2017, *Anopheles gambiae* (*s.l*.) larvae were sampled in or around the nine vector surveillance sentinel sites of the Gambia National Malaria Control Programme (GNMCP) and in a few additional sampling points. Using WHO susceptibility bioassays, female adult mosquitoes were exposed to insecticide-impregnated papers. Molecular identification of sibling species and insecticide resistance molecular markers was done on a subset of 2000 female mosquitoes.

**Results:**

A total of 4666 wild-caught female adult mosquitoes were exposed to either permethrin (*n* = 665), deltamethrin (*n* = 744), DDT (*n* = 1021), bendiocarb (*n* = 990) or pirimiphos-methyl (*n* = 630) insecticide-impregnated papers and control papers (*n* = 616). Among the 2000 anophelines, 1511 (80.7%) were *Anopheles arabiensis*, 204 (10.9%) *Anopheles coluzzii*, 75 (4%) *Anopheles gambiae* (*s.s.*), and 83 (4.4%) *An. gambiae* (*s.s.*) and *An. coluzzii* hybrids. There was a significant variation in the composition and species distribution by regions and year, *P* = 0.009. Deltamethrin, permethrin and DDT resistance was found in *An. arabiensis,* especially in the coastal region, and was mediated by *Vgsc-1014F/S* mutations (odds ratio = 34, *P* = 0.014). There was suspected resistance to pirimiphos-methyl (actellic 300CS) in the North Bank Region although only one survivor had the *Ace-1*-119S mutation.

**Conclusions:**

As no confirmed resistance to bendiocarb and actellic 300CS was detected, the national malaria control programme can continue using these insecticides for IRS. Nevertheless, the detection of *Ace-1 119S* mutation warrants extensive monitoring. The source of insecticide pressure driving insecticide resistance to pyrethroids and DDT detected at the coastal region should be further investigated in order to properly manage the spread of resistance in The Gambia.

**Electronic supplementary material:**

The online version of this article (10.1186/s13071-019-3538-0) contains supplementary material, which is available to authorized users.

## Background

Malaria control in The Gambia as in other endemic countries relies on vector control with long-lasting insecticidal nets (LLIN) and indoor residual spraying (IRS) [1]; globally, these two interventions have averted about 78% of malaria-related deaths and morbidity [2]. Nevertheless, as they rely on efficacious insecticides, the emergence and spread of insecticide resistance may represent a major threat [3–5], although LLINs would still offer personal protection against mosquito bites [6].

IRS has been implemented in The Gambia since 2008, and the insecticide employed for this purpose has varied over the years, firstly DDT and then bendiocarb in 2015–2016, after significant resistance to DDT was observed [4, 7], and pirimiphos-methyl (actellic 300CS) since 2017. Changing the insecticide used for IRS follows the recommendations of the World Health Organization (WHO) Global Plan for Insecticide Resistance Management (GPIRM) of regularly rotating insecticides of different classes [8].

The choice of the insecticide for use in IRS should be based on the susceptibility of the local vectors to the WHO approved insecticides [9]. The Gambia has routinely conducted insecticide susceptibility tests to guide IRS campaigns (GNMCP unpublished reports). The most recent country-wide survey was done in 2013–2014 and reported high levels of resistance to DDT and pyrethroids [4].

In collaboration with the Gambian National Malaria Control Programme (GNMCP), the susceptibility of the local malaria vectors to the insecticides commonly used for vector control, namely deltamethrin, permethrin, DDT, bendiocarb and actellic 300CS, was determined during the 2016 and 2017 malaria transmission seasons.

## Methods

### Study area

**Fig. 1:**
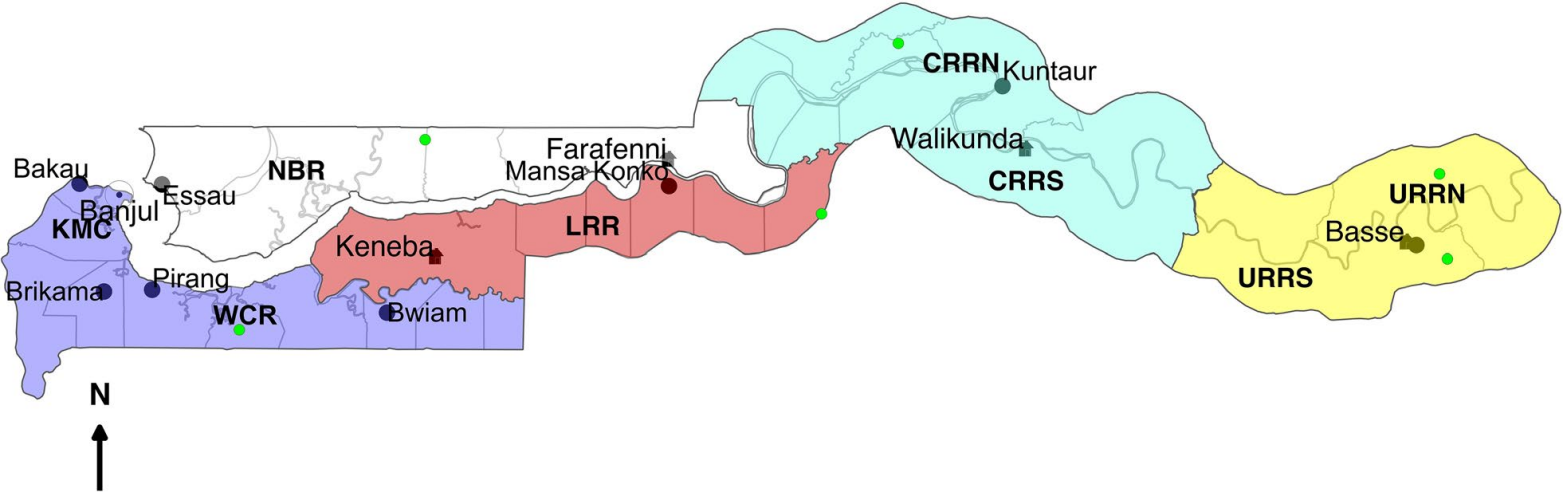
The map of The Gambia showing the five administrative regions (coloured areas) and vector surveillance sentinel sites. *Abbreviations*: KMC, Kanifing Municipality; WCR, West Coast Region; NBR, North Bank Region; LRR, Lower River Region; CRR(N/S), Central River Region North/South; URR(N/S), Upper River Region North/South. Green dots represent additional sampling points from the 2013–2014 study [4]

This is a nationwide study carried out in villages at 9 GNMCP vector surveillance sentinel sites: Essau, Farafenni, Mansa Konko, Kuntaur, Brikama, Basse, Bwiam, Pirang, Bakau. Additional sites, Besse, Yallal Ba, Chogen Wellingara, Wali Kunda, Gambisarra, Gunjur Koto and Sare Wuro, that formed part of a countrywide insecticide susceptibility study in 2013–2014, were also included to provide a robust map of insecticide susceptibility patterns [4] (Additional file 1: Table S1). Villages were grouped into 7 regions to provide an overview of susceptibility per region (Fig. 1). Kanifing Municipality (KMC) located in West Coast Region (WCR) and representing an urban settlement, was considered a region on its own. Central River Region (CRR) was also divided into North (CRRN) and South (CRRS) bank. Similarly, Upper River Region (URR) was divided into North (URRN) and South (URRS). North Bank Region (NBR) was taken as one region.

### Collection and rearing of larvae

Malaria vectors were collected over two malaria transmission seasons (July-December), in 2016 and 2017. Two mosquito collection strategies were used to generate adults for susceptibility tests following the WHO susceptibility bioassay protocol [10], namely larva sampling in their natural breeding habitats or (when larvae could not be found) collection of blood-fed or gravid adults.

*Anopheles gambiae* (*s.l.*) larvae were collected from breeding sites in all but two villages, Ngedden and Sare Seedy, where blood-fed or gravid mosquitoes were sampled using mouth aspirators. The larvae were reared according to standard conditions of 27 ± 2 °C and relative humidity of 80 ± 5% in the insectaries at the MRC field stations in Basse and Wali Kunda. The blood-fed/gravid females were placed in a cage and induced to lay eggs by placing a dump filter paper in a Petri dish. They were fed on 10% sugar solution until no more eggs were laid. The eggs were reared to larvae under similar conditions as larvae collected from the field until adults emerged.

### Insecticide susceptibility bioassays of *Anopheles gambiae* (*s.l.*)

Insecticide-impregnated papers were prepared and supplied by Liverpool Insecticide Testing Establishment (LITE) at the Liverpool School of Tropical Medicine, Liverpool, UK. To validate the effectiveness of the WHO test papers, susceptible laboratory mosquito colony (*Anopheles coluzzii* Yaoundé strain) was exposed to all the impregnated and control papers. Groups of 20–25 three- to five-days-old adult female mosquitoes were exposed for an hour to either 4% DDT, 0.25% pirimiphos-methyl, 0.1% bendiocarb, 0.75% permethrin and 0.05% deltamethrin impregnated papers [10]. For each insecticide class exposure experiment, random samples of field-collected and susceptible laboratory strain were separately exposed to appropriate control papers. Mortality 24 h post-exposure was recorded for each replicate experiment. All exposure experiments were performed under the temperature of 26 ± 2 °C and relative humidity of 70 ± 10%.

Since mosquito density was low, it was not possible to perform the recommended 5 replicates per insecticide per village. Therefore, to increase the power of the mortality estimates for each insecticide, village data were pooled according to geographical location into 7 regions (Fig. 1, Additional file 1: Table S2). The temperature and humidity were also recorded during the exposure periods.

All exposed mosquitoes were stored in labelled individual Eppendorf tubes with a desiccant-silica gel. They were then transported to the coast for molecular genotyping at the MRC to determine species [11, 12]; known insecticide resistance mutations at the *Voltage-gated sodium channel* (*Vgsc*-1014F and *Vgsc*-1014S) gene that confer *knockdown resistance* (*kdr*) against DDT and pyrethroids and mutation at the *Ace-1* (*Ace-1-119S*) gene conferring resistance to organophosphates and carbamates were also investigated using Taqman polymerase chain reactions (PCR) [13, 14]. This was done in a subset of 2000 mosquitoes randomly selected according to 4 main criteria: insecticide exposed; phenotypic status (dead or alive); year of collection and lastly, the number of samples selected per region was proportional to the number of mosquitoes collected (Additional file 1: Table S3).

### Statistical analysis

All statistical analyses were performed in R statistical package [15]. Mortality was estimated by dividing the number of dead mosquitoes per replicate 24 h post-exposure by the total number of mosquitoes exposed. Spearman’s correlation was used to test the effect of temperature and humidity on mortality. Odds ratios, Chi-square and Fisherʼs exact tests were used to measure the association between resistance mutations and susceptibility to insecticides. Proportionality tests (tests of confidence intervals) [16] were used to establish differences in mortality between regions for each of the insecticides tested. General linear models (GLM) with logit link function for a binomial dependent variable was used to model the effect of *kdr*, insecticide, species, region and year on mortality.

## Results

A total of 4666 female *An. gambiae* (*s.l.*) were successfully reared and tested for phenotypic resistance. Of these, 665 were tested against permethrin, 744 to deltamethrin-, 1021 to DDT-, 990 to bendiocarb-, 630 to actellic 300CS-impregnated papers and 616 to control papers. In addition, 600 laboratory strains from Yaoundé, Cameroon, were also used as controls during exposure bioassays.

### Species composition and distribution

**Fig. 2:**
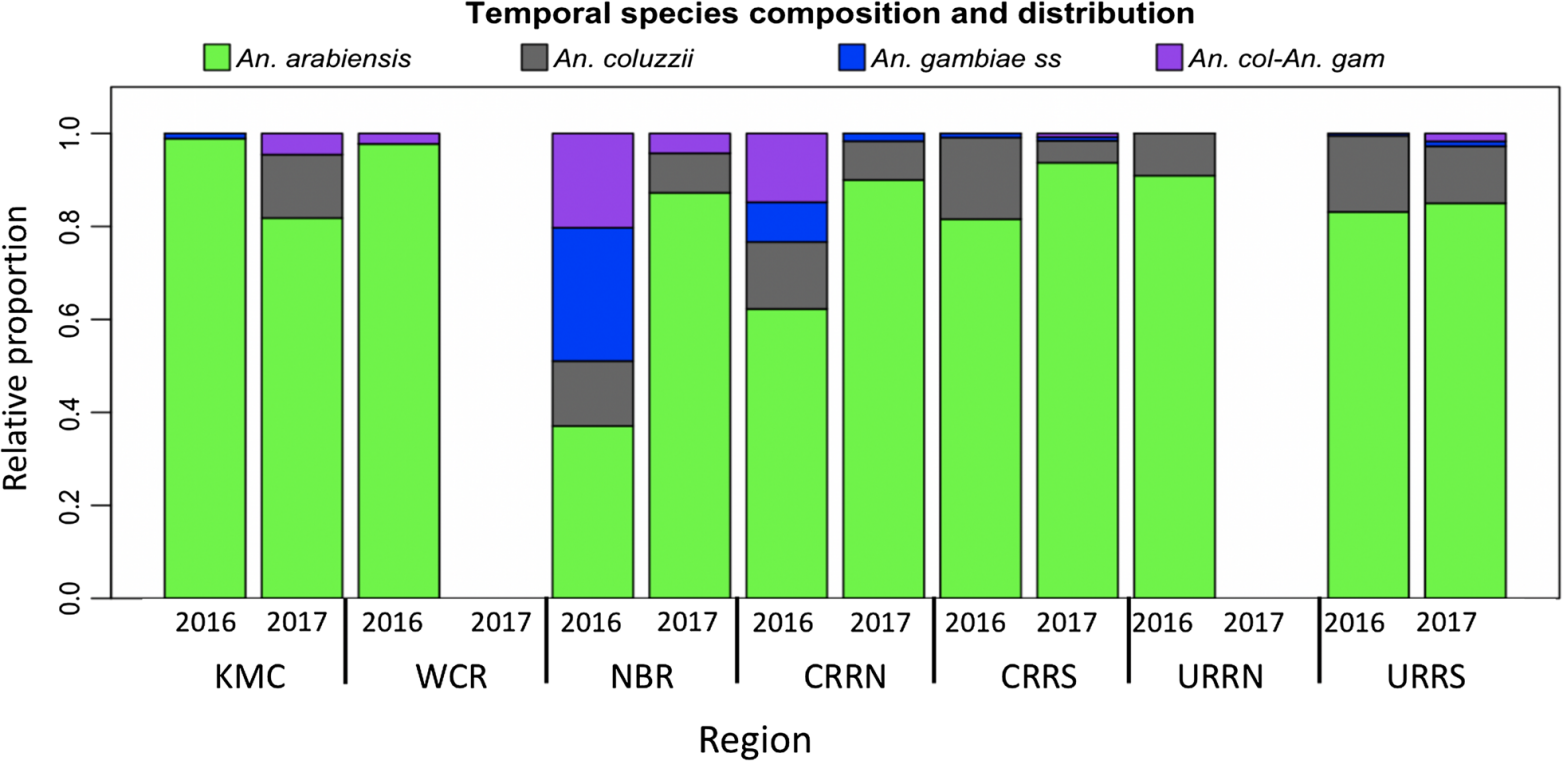
Species distribution and composition of *Anopeheles gambiae* (*s.l.*) collected during the 2016 and 2017 transmission seasons from GNMCP sentinel sites in The Gambia. *Abbreviations*: KMC, Kanifing Municipality; WCR, West Coast Region; NBR, North Bank Region; LRR, Lower River Region; CRR(N/S), Central River Region North/South; URR(N/S), Upper River Region North/South

Of the 2000 anophelines screened for sibling species and molecular markers of insecticide resistance, 1511 (80.7%) were *Anopheles arabiensis*, 204 (10.9%) *Anopheles coluzzii*, 75 (4%) *Anopheles gambiae* (*s.s.*) and 83 (4.4%) hybrid between *An. gambiae* (*s.s.*) and *An. coluzzii* (*An. gam-An. col*). The composition and distribution of these species varied by region and year (Fig. 2, *P* = 0.009). Most *An. gambiae* (*s.s.*) were collected in 2016 in the NBR and CRR North; in 2017 few *An. gambiae* (*s.s.*) were collected.

### *Anopheles gambiae* (*s.l.*) phenotypic resistance to insecticides

All susceptible laboratory strain mosquitoes died upon exposure to insecticide-impregnated papers except those in the control papers. During the exposure experiments, mortality in the control group was always less than 5% (data not shown) and therefore Abbottʼs correction was not applied to the mortality estimates. There was no effect of temperature and humidity on mortality, *P* = 0.299.

#### Deltamethrin and permethrin

**Fig. 3:**
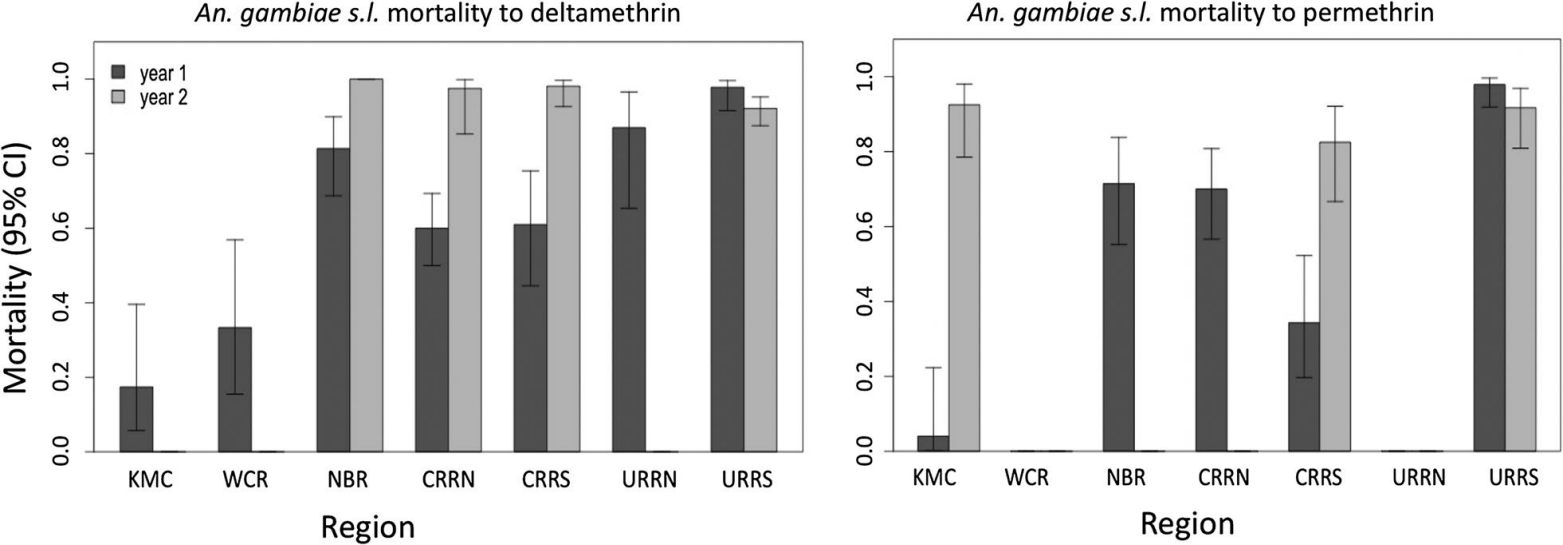
Susceptibility by region of *An. gambiae* (*s.l.*) to deltamethrin and permethrin sampled during the 2016 and 2017 malaria transmission seasons in The Gambia. Missing bars represent missing data where no test was done. *Abbreviations*: KMC, Kanifing Municipality; WCR, West Coast Region; NBR, North Bank Region; LRR, Lower River Region; CRR(N/S), Central River Region North/South; URR(N/S), Upper River Region North/South

There was marked resistance to deltamethrin and permethrin, especially in KMC and WCR in the coastal region, with only about 30% mortality (Fig. 3). Resistance was also observed in NBR and CRR in 2016 but not in 2017 (Fig. 3). In URRS, resistance to pyrethroids was low in both years with mortality rate of 97%. Permethrin was not tested in 2017 in WCR and NBR. There was high Spearmanʼs correlation coefficient (*r* = 0.89, *P* = 0.03) between deltamethrin and permethrin susceptibility. Paired and unpaired Mann-Whitney U-tests did not detect differences in mortalities between the two insecticides.

#### DDT

**Fig. 4:**
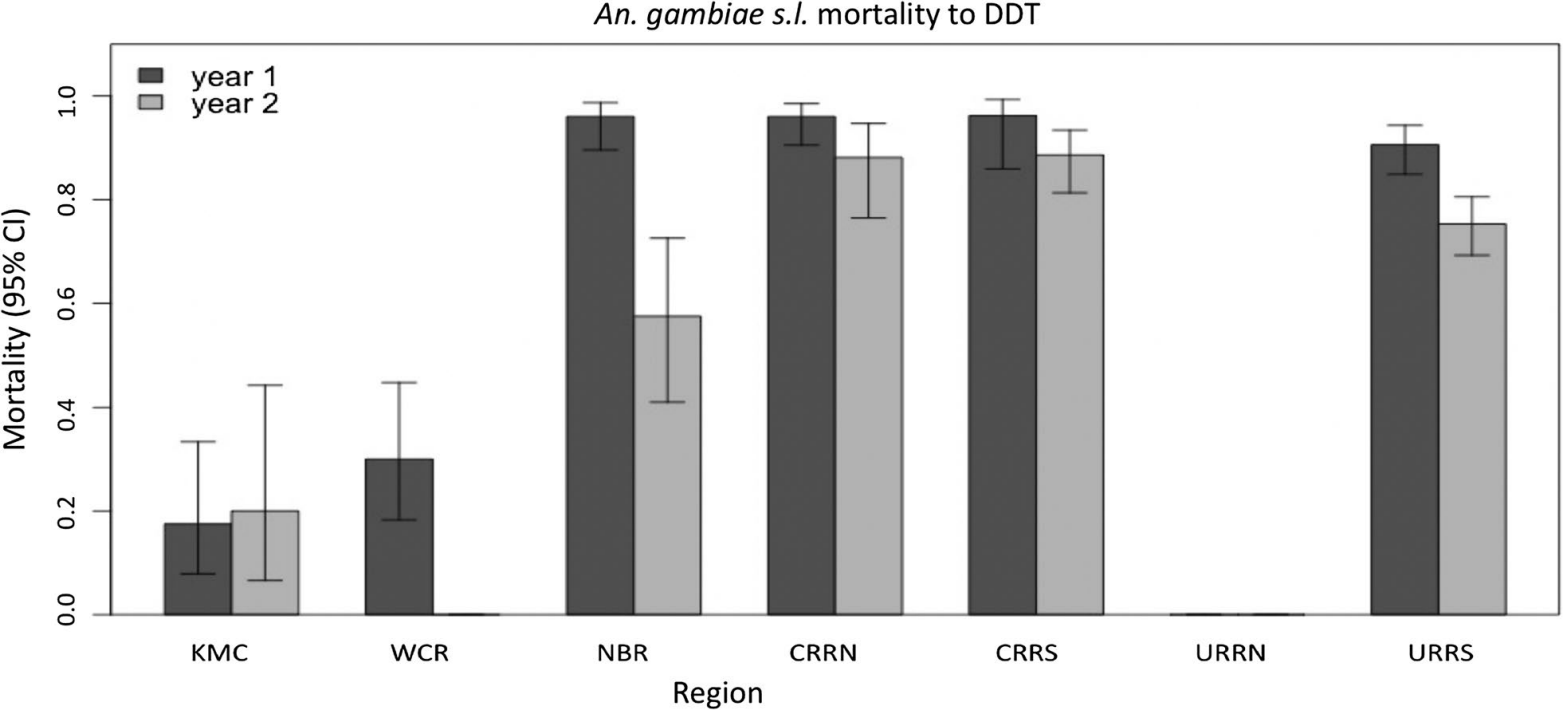
Susceptibility by region of *An. gambiae* (*s.l.*) to DDT sampled during the 2016 and 2017 rainy seasons in The Gambia. Missing bars represent missing data where no test was done. *Abbreviations*: KMC, Kanifing Municipality; WCR, West Coast Region; NBR, North Bank Region; LRR, Lower River Region; CRR(N/S), Central River Region North/South; URR(N/S), Upper River Region North/South

Higher phenotypic resistance to DDT was recorded in KMC and WCR in the coastal regions, with mortality of about 30% (Fig. 4). In NBR, resistance was low in 2016, with 96% mortality but mortality was significantly lower in 2017, 57.5% (*P* < 0.001). In CRRS, CRRN and URRS, resistance was only suspected in 2016; however, in 2017 resistance in URRS increased significantly, *P* = 0.0001 (Fig. 3).

#### Bendiocarb and pirimiphos-methyl

**Fig. 5:**
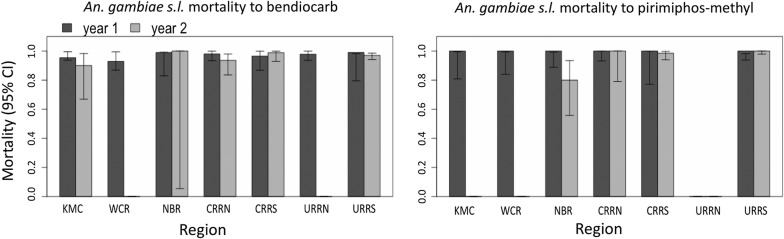
Susceptibility by region of *An. gambiae* (*s.l.*) to bendiocarb and pirimiphos-methyl sampled during the transmission seasons of 2016 and 2017 in The Gambia. Missing bars represent missing data where no test was done. *Abbreviations*: KMC, Kanifing Municipality; WCR, West Coast Region; NBR, North Bank Region; LRR, Lower River Region; CRR(N/S), Central River Region North/South; URR(N/S), Upper River Region North/South

Mortality estimates against bendiocarb (carbamate) and pirimiphos-methyl (organophosphate) indicate that these insecticides are still effective against malaria vectors (Fig. 5). However, there was suspected emerging resistance to bendiocarb in KMC, WCR, CRRS and URRN; in NBR suspected emerging resistance to actellic 300CS was observed in 2017 (Fig. 4).

### Frequency of insecticide resistance molecular markers

Of the 2000 *An. gambiae* (*s.l.*) screened for sibling species and markers of insecticide resistance, 93.65% (1873) were successfully genotyped for resistance mutations. Three mosquitoes (2 from CRRN and one from NBR) had the *Ace-1 G119S* mutation but only one survived exposure to carbamates and no further statistical testing was done.

The frequency of the *Vgsc-L1014F* (*kdr*F) and *Vgsc-L1014S* (*kdr*S) alleles varied by species and region, with the latter significantly higher than the former, *P* < 0.001 (Additional file 1: Figure S1 and Table S4). The *kdrS* and *kdrF* frequencies were significantly higher at the coast (WCR and KMC) than in the other regions (*P* < 0.001). In *An. arabiensis*, the frequency of the *kdr* alleles varied significantly by region, *P* < 0.01. Low numbers for other species precluded statistical estimation of frequency and confidence intervals around the estimates (Additional file 1: Table S4).

#### *Knockdown resistance* (*kdr*) markers and phenotypic resistance

In WCR and KMC, *An. arabiensis* with the *kdr* mutations were more likely to survive after exposure to either pyrethroids or DDT in both 2016 (odds ratio, OR = 34, *P* = 0.014) and 2017, (OR = 2.5, *P* < 0.025). In URR, *kdrS* conferred survival advantage for *An. arabiensis* exposed to pyrethroids/DDT only when the data from the two years were pooled (OR = 2.5, *P* = 0.003). In CRR, there was no relationship between survival and any of the *kdr* mutations.

In a generalized linear model (GLM), *kdrF/S*, together with region and year explained significant variation in mortality estimates of *An. gambiae* (*s.l.*) to pyrethroids and DDT (Table 1). Although species was not significantly associated with mortality, the best-fit model was the one including species as a variable. Including insecticide did not significantly improve the model, and this variable was excluded. Both *kdrF* and *kdrS* significantly explained survival of mosquitoes against the pyrethroids and DDT (Table 2).

**Table 1:** Effects of region, species, *kdr* and year on mortality of mosquitoes to deltamethrin, permethrin and DDT using GLM

Factor	*df*	Deviance	Residual *df*	Residual deviance	*P-*value
Region	6	175.68	1147	1344.15	< 0.001
Species	3	3.97	1144	1340.18	0.135
*kdr*	5	46.17	1139	1294.00	< 0.001
Year	1	7.33	1138	1286.68	< 0.01

*Abbreviation*: df, degrees of freedom

**Table 2:** Generalized linear model testing the effects of region, species, *kdr* and year on mortality of mosquitoes to deltamethrin, permethrin and DDT

Factor	Factor level	Estimate	SE	*z*-value	*P*-value
	Intercept	− 0.78	0.29	− 2.69	0.007
Region	CRR North	− 0.02	0.25	− 0.09	0.926
	CRR South	− 0.06	0.27	− 0.23	0.818
	KMC	1.34	0.33	4.00	< 0.001
	URR North	− 0.94	0.73	− 1.29	0.198
	URR South	− 0.76	0.26	− 2.95	0.003
	WCR	1.43	0.45	3.20	0.001
Species	Ar	0.25	0.23	1.06	0.290
	MS	− 0.37	0.4	− 0.94	0.349
	S	− 0.52	0.48	− 1.08	0.280
*kdr*	FF	1.79	0.44	4.07	< 0.001
	FS	0.87	0.28	3.14	0.002
	LF	0.42	0.24	1.72	0.086
	LS	0.01	0.19	0.05	0.957
	SS	1.35	0.26	5.10	< 0.001
Year	2017	− 0.4	0.15	− 2.71	0.007

*Abbreviation*: SD, standard deviation

## Discussion

In The Gambia, malaria vectors are still susceptible to bendiocarb and actellic 300CS although one *An. arabiensis* from CRRN and two *An. gam-An. col* hybrids from CRRN and NBR had the *Ace-1 119S* mutation that confers resistance to organophosphates and carbamates. Conversely, resistance to pyrethroids and DDT, mediated by *Vgsc-L1014F* and *Vgsc-L1014S*, was widespread and more frequent in the coastal region in western Gambia.

Consistent with other published reports [4, 17, 18], three members of the *An. gambiae* complex, namely *An. gambiae* (*s.s.*), *An. arabiensis* and *An. coluzzii*, including *An. gam*-*An. col* hybrids, were identified and their distribution varied substantially by region; *An. arabiensis* was the dominant species in almost all regions while *An. gambiae* (*s.s.*) was found mainly in two regions. Such results differ from those of the latest nationwide entomological study carried out in 2013–2014 as *An. gambiae* (*s.s.*) and *An. coluzzii* were much more frequent at that time [4].

Such change in species composition may be due to the use of bendiocarb and actellic 300CS for IRS, which killed the more anthropogenic and endophilic *An. gambiae* (*s.s.*) and *An. coluzzii*, increasing the proportion of the more zoophilic *An. arabiensis*. This change in species composition has also been observed in Kenya, Tanzania and Senegal [19–21]. The predominance of *An. arabiensis* compared to other, more efficient vectors such as *An. gambiae* (*s.s.*), could have also contributed to the decline in malaria transmission observed in The Gambia over the last few years.

Insecticide resistance is known to vary among species and in time and changes in species composition may impact on the status of insecticide resistance [4]. Differences in species composition in NBR could have led to observed variation in observed insecticide resistance.

### Mechanisms of insecticide resistance

*Vgsc-1014F* and *Vgsc-1014S* mutations in *An. arabiensis* were associated with phenotypic resistance, particularly in WCR and KMC. This differs from previous reports from The Gambia in which the lack of association between mutations and resistance in this species was explained by the few *An. arabiensis* tested and the lack of *kdr* survivors [4, 22]. However, in neighbouring Senegal, *kdr* alleles in *An. arabiensis* conferred phenotypic resistance to pyrethroids and DDT, confirming the present results [23, 24].

Among the mosquitoes that survived exposure to pyrethroids/DDT, there were some without *kdr* mutations, possibly indicating other mechanisms of resistance such as metabolic resistance already implicated in conferring resistance in *An. gambiae* (*s.s.*) but not in *An. arabiensis* [4]. In neighboring Senegal, metabolic and target site resistance have been implicated in *An. arabiensis* resistance to pyrethroids and DDT [23].

Both bendiocarb and actellic 300CS insecticides are effective against *An. arabiensis* in The Gambia. For the suspected resistance to actellic 300CS and bendiocarb, only three mosquitoes carried the *Ace-1 119S* mutation and only one *An. gam-An. col* hybrid survived exposure to bendiocarb. One *An. arabiensis* and one *An. gam-An. col* hybrid died upon exposure to bendiocarb. Although the *Ace-1 119S* mutation was not found in samples from the URR, it had been previously identified in *An. gambiae* (*s.s.*) from the URR [4]. This may indicate that continued use of bendiocarb/actellic 300CS for IRS may increase its frequency, requiring continuous monitoring of the frequency of this mutation.

As malaria continues to decline, urban malaria will pose challenges to NMCPs in SSA [25]. The Gambia is preparing to enter into the pre-elimination phase by 2020. However, the goal might be threatened by the high insecticide resistance in the western part of the country, which is mostly urban. In this region, the GNMCP distribute only LLIN while IRS is not implemented. The use of commercial pesticides for domestic use and in agriculture is known to contribute to development of insecticide resistance [26, 27]. There is little information on the actual use of pesticides in this area and the GNMCP is collecting such information to identify other sources of insecticide resistance pressure.

Despite the observed insecticide resistance to pyrethroids, LLINs continue to offer personal protection against mosquito bites [6] and should still be used for malaria control. The use of nets containing piperonyl butoxide (PBO) and insecticide can be useful for restoring the efficacy of insecticides used in LLINs where metabolic resistance is present [28, 29]. Additionally, targeting juvenile stages of mosquitoes using larvicides can help existing vector control activities to maintain the gains made in malaria control. For IRS, resistance mutations should be monitored closely to avert operational failures in malaria control [30–32].

## Conclusions

Insecticide resistance against deltamethrin, permethrin and DDT mediated by *Vgsc-1014F/S* was present in *An. arabiensis*, especially in the coastal region. Between 2016 and 2017, *An. arabiensis* was the main malaria vector in the Gambia. There was no resistance to bendiocarb across the country but for pirimiphos-methyl, there was suspected resistance only in NBR in 2017. However, the detection of *Ace-1 119S* warrants extensive monitoring to ensure it does not spread across the country. The GNMCP can therefore continue using pirimiphos-methyl for IRS while adhering to the recommendations of the Global Plan for Insecticide Resistance Management. The GNMCP should also consider doing further studies in the coastal region to understand the source of insecticide pressure driving resistance in this setting.

## Additional files

**Additional file 1: Table S1.** Villages and respective regions where *Anopheles gambiae* mosquitoes were sampled in 2016 and 2017 malaria transmission seasons. **Table S2**. Number of mosquito exposure replicates per insecticide by region. **Table S3**. The distribution and number of mosquitoes screened for molecular markers of insecticide resistance by year. **Figure S1**. Frequency of *kdr* alleles in *An. gambiae* (*s.l.*) sampled during the transmission seasons of 2016 and 2017 in The Gambia and exposed to either deltamethrin, permethrin or DDT. Abbreviations denote administrative regions in The Gambia as in Fig. 1. **Table S4**. Frequency of *kdr* alleles, L1014F and L1014S per species by region.

**Additional file 2.** Raw dataset of the WHO insecticide susceptibility tests carried out during the 2016 and 2017 malaria transmission seasons in The Gambia.

## Data Availability

Data supporting the conclusions of this article are included within the article and its additional files. The raw data are provided in Additional file 2.

## Abbreviations

LLINS: long-lasting insecticidal bednets
IRS: indoor residual spraying
GNMCP: Gambia National Malaria Control Programme
DDT: dichlorodiphenyltrichloroethane
GLM: general linear models
kdr: knockdown resistance
Vgsc: voltage-gated sodium channel gene
Gste2: glutathione S-transferase E2
Ace1: acetylcholinesterase
KMC: Kanifing Municipality
WCR: West Coast Region
CRRN: Central River Region North
CRRS: Central River Region South
URRN: Upper River Region North
URRS: Upper River Region South
NBR: North Bank Region
