# Supplementary material for: Status of insecticide resistance in Anopheles gambiae (s.l.) of The Gambia
Source: Parasit Vectors. 2019 Jun 4;12:287. doi: 10.1186/s13071-019-3538-0 (PMC6549352; doi:10.1186/s13071-019-3538-0)
Supplement: Supplementary file 1 — Additional file 1: Table S1. Villages and respective regions where Anopheles gambiae mosquitoes were sampled in 2016 and 2017 malaria transmission seasons. Table S2. Number of mosquito exposure replicates per insecticide by region. Table S3. The distribution and number of mosquitoes screened for molecular markers of insecticide resistance by year. Figure S1. Frequency of kdr alleles in An. gambiae (s.l.) sampled during the transmission seasons of 2016 and 2017 in The Gambia and exposed to either deltamethrin, permethrin or DDT. Abbreviations denote administrative regions in The Gambia as in Fig. 1. Table S4. Frequency of kdr alleles, L1014F and L1014S per species by region. [file 13071_2019_3538_MOESM1_ESM.docx]

Additional file 1: TableS1. Villages and respective regions where *Anopheles gambiae* mosquitoes were sampled in 2016 and 2017 malaria transmission seasons.

| **Region** | **village** |
| --- | --- |
| Kanifing Municipality (KMC) | Bakau |
|  | Taling Ding |
|  | Serre Kunda |
|  | Ghana town |
|  | Lamin |
| West Coast Region (WCR) | Bwiam |
|  | Besse |
|  | Brikama |
|  | Pirang |
|  | Bulock |
| North Bank Region (NBR) | Essau |
|  | Yallal Ba |
|  | Chogen Wellingara |
|  | Njamba kunda |
|  | Farafenni |
| Central River Region North (CRRN) | Sare Seedy |
|  | Ngedden |
|  | Madina |
|  | Kuntaur |
|  | Barajali |
|  | Wassu |
| Central River Region South (CRRS) | Jakhaly |
|  | Brikama Ba |
|  | Saruja |
|  | Wali Kunda |
|  | Boiram |
|  | Taifa |
| Upper River Region North (URRN) | Gunjur Koto |
|  | Sare Wuro |
|  | Madina Koto |
| Upper River Region South (URRS) | Basse |
|  | Gambisara |
|  | Sare Alpha |
|  | Simoto Tuba |
|  | Chamoi |

Additional file 1: Table S2. Number of mosquito exposure replicates per insecticide by region.

| **Region** | **Deltamethrin** | **Permethrin** | **Bendiocarb** | **Pirimophos methyl** | **DDT** | **OP-control** | **PY-Control** | **Total tests** |
| --- | --- | --- | --- | --- | --- | --- | --- | --- |
| KMC | 2 | 3 | 4 | 1 | 3 | 2 | 1 | 16 |
| WCR | 1 | 0 | 2 | 1 | 2 | 1 | 1 | 8 |
| NBR | 5 | 3 | 6 | 3 | 7 | 1 | 2 | 27 |
| CRR North | 3 | 4 | 5 | 2 | 5 | 1 | 2 | 22 |
| CRR South | 9 | 11 | 9 | 9 | 13 | 2 | 2 | 55 |
| URR North | 3 | 0 | 4 | 0 | 3 | 0 | 0 | 10 |
| URR South | 15 | 8 | 17 | 11 | 18 | 5 | 3 | 77 |
| **Total tests** | 38 | 29 | 47 | 27 | 51 | 12 | 11 | 215 |

Additional file 1: Table S3. The distribution and number of mosquitoes screened for molecular markers of insecticide resistance by year.

|  |  | Insecticide | | | | | | | | | |
| --- | --- | --- | --- | --- | --- | --- | --- | --- | --- | --- | --- |
|  |  | Deltamethrin | | Permethrin | | DDT | | Bendiocarb | | Pirimiphos-  Methyl | |
|  |  | 2016 | 2017 | 2016 | 2017 | 2016 | 2017 | 2016 | 2017 | 2016 | 2017 |
| Phenotype | Alive | 103 | 17 | 76 | 40 | 94 | 106 | 13 | 16 | 0 | 6 |
|  | Dead | 92 | 126 | 65 | 147 | 188 | 141 | 201 | 178 | 103 | 161 |
|  | Total | 195 | 143 | 141 | 187 | 282 | 247 | 214 | 194 | 103 | 167 |


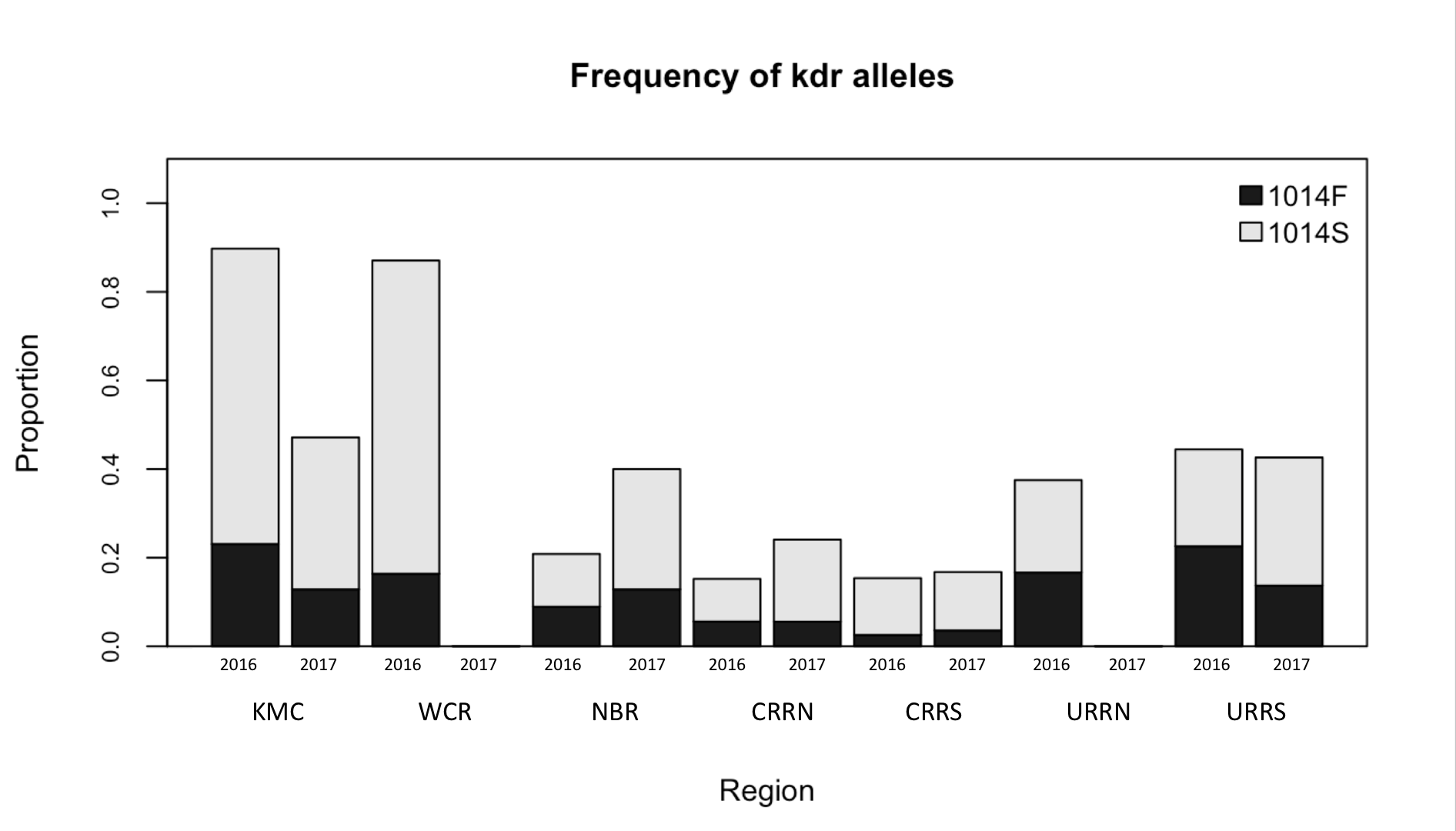


Additional file 1: FigureS1. Frequency of *kdr* alleles in *An. gambiae* sl sampled during the transmission seasons of 2016 and 2017 in The Gambia and exposed to either deltamethrin, permethrin or DDT. Abbreviations denote administrative regions in The Gambia as in Fig. 1.

Additional file 1: TableS4: Frequency of *kdr* alleles, L1014F and L1014S per species by region

| **Region** | **species** | **FF** | **FS** | **LF** | **LL** | **LS** | **SS** | **S** | **F** | **total** | **F_freq** | **F_lower** | **F_upper** | **S_freq** | **S_lower** | **S_upper** |
| --- | --- | --- | --- | --- | --- | --- | --- | --- | --- | --- | --- | --- | --- | --- | --- | --- |
| CRRN | *An. arabiensis* | 1 | 9 | 9 | 109 | 42 | 5 | 61 | 20 | 350 | 0.057 | 0.036 | 0.088 | 0.174 | 0.137 | 0.219 |
| CRRN | *An. coluzzii* | 0 | 0 | 2 | 31 | 0 | 0 | 0 | 2 | 66 | 0.030 | 0.005 | 0.115 | 0.000 | 0.000 | 0.069 |
| CRRN | *An gam-An col* hybrid | 0 | 0 | 2 | 20 | 0 | 0 | 0 | 2 | 44 | 0.045 | 0.008 | 0.167 | 0.000 | 0.000 | 0.100 |
| CRRN | *An. gambiae* ss | 0 | 0 | 3 | 9 | 0 | 0 | 0 | 3 | 24 | 0.125 | 0.033 | 0.335 | 0.000 | 0.000 | 0.172 |
| CRRS | *An. arabiensis* | 0 | 2 | 11 | 156 | 51 | 5 | 63 | 13 | 450 | 0.029 | 0.016 | 0.050 | 0.140 | 0.110 | 0.176 |
| CRRS | *An. coluzzii* | 0 | 0 | 3 | 16 | 1 | 0 | 1 | 3 | 40 | 0.075 | 0.020 | 0.215 | 0.025 | 0.001 | 0.147 |
| CRRS | *An gam-An col* hybrid | 0 | 0 | 0 | 0 | 0 | 0 | 0 | 0 | 0 | 0.000 | 0.000 | 0.000 | 0.000 | 0.000 | 0.000 |
| CRRS | *An. gambiae* ss | 0 | 0 | 0 | 0 | 0 | 0 | 0 | 0 | 0 | 0.000 | 0.000 | 0.000 | 0.000 | 0.000 | 0.000 |
| KMC | *An. arabiensis* | 6 | 27 | 4 | 16 | 12 | 43 | 125 | 43 | 216 | 0.199 | 0.149 | 0.260 | 0.579 | 0.510 | 0.645 |
| KMC | *An. coluzzii* | 0 | 0 | 1 | 1 | 1 | 0 | 1 | 1 | 6 | 0.167 | 0.009 | 0.635 | 0.167 | 0.009 | 0.635 |
| KMC | *An gam-An col* hybrid | 0 | 0 | 1 | 0 | 0 | 0 | 0 | 1 | 2 | 0.500 | 0.095 | 0.905 | 0.000 | 0.000 | 0.802 |
| KMC | *An. gambiae* ss | 0 | 0 | 0 | 0 | 0 | 1 | 2 | 0 | 2 | 0.000 | 0.000 | 0.802 | 1.000 | 0.198 | 1.000 |
| NBR | *An. arabiensis* | 1 | 4 | 5 | 26 | 19 | 7 | 37 | 11 | 124 | 0.089 | 0.047 | 0.157 | 0.298 | 0.221 | 0.388 |
| NBR | *An. coluzzii* | 2 | 0 | 1 | 12 | 2 | 0 | 2 | 5 | 34 | 0.147 | 0.055 | 0.318 | 0.059 | 0.010 | 0.211 |
| NBR | *An gam-An col* hybrid | 0 | 0 | 7 | 16 | 0 | 0 | 0 | 7 | 46 | 0.152 | 0.068 | 0.295 | 0.000 | 0.000 | 0.096 |
| NBR | *An. gambiae* ss | 0 | 0 | 1 | 16 | 0 | 0 | 0 | 1 | 34 | 0.029 | 0.002 | 0.171 | 0.000 | 0.000 | 0.126 |
| URRN | *An. arabiensis* | 0 | 0 | 2 | 5 | 3 | 1 | 5 | 2 | 22 | 0.091 | 0.016 | 0.306 | 0.227 | 0.087 | 0.458 |
| URRN | *An. coluzzii* | 1 | 0 | 0 | 0 | 0 | 0 | 0 | 2 | 2 | 1.000 | 0.198 | 1.000 | 0.000 | 0.000 | 0.802 |
| URRN | *An gam-An col* hybrid | 0 | 0 | 0 | 0 | 0 | 0 | 0 | 0 | 0 | 0.000 | 0.000 | 0.000 | 0.000 | 0.000 | 0.000 |
| URRN | *An. gambiae* ss | 0 | 0 | 0 | 0 | 0 | 0 | 0 | 0 | 0 | 0.000 | 0.000 | 0.000 | 0.000 | 0.000 | 0.000 |
| URRS | *An. arabiensis* | 7 | 35 | 34 | 116 | 73 | 40 | 188 | 83 | 610 | 0.136 | 0.110 | 0.166 | 0.308 | 0.272 | 0.347 |
| URRS | *An. coluzzii* | 6 | 2 | 17 | 28 | 2 | 0 | 4 | 31 | 110 | 0.282 | 0.202 | 0.377 | 0.036 | 0.012 | 0.096 |
| URRS | *An gam-An col* hybrid | 3 | 0 | 0 | 0 | 0 | 0 | 0 | 6 | 6 | 1.000 | 0.517 | 1.000 | 0.000 | 0.000 | 0.483 |
| URRS | *An. gambiae* ss | 2 | 0 | 0 | 0 | 0 | 0 | 0 | 4 | 4 | 1.000 | 0.396 | 1.000 | 0.000 | 0.000 | 0.604 |
| WCR | *An. arabiensis* | 2 | 14 | 1 | 0 | 14 | 27 | 82 | 19 | 116 | 0.164 | 0.104 | 0.247 | 0.707 | 0.614 | 0.786 |
| WCR | *An. coluzzii* | 0 | 0 | 0 | 0 | 0 | 0 | 0 | 0 | 0 | 0.000 | 0.000 | 0.000 | 0.000 | 0.000 | 0.000 |
| WCR | *An gam-An col* hybrid | 0 | 0 | 0 | 0 | 0 | 0 | 0 | 0 | 0 | 0.000 | 0.000 | 0.000 | 0.000 | 0.000 | 0.000 |
| WCR | *An. gambiae* ss | 0 | 0 | 0 | 0 | 0 | 0 | 0 | 0 | 0 | 0.000 | 0.000 | 0.000 | 0.000 | 0.000 | 0.000 |
